# Highly Efficient Synthesis of Rare Sugars from Glycerol in Endotoxin-Free *ClearColi* by Fermentation

**DOI:** 10.3390/foods12163078

**Published:** 2023-08-16

**Authors:** Yahui Gao, Zhou Chen, Hideki Nakanishi, Zijie Li

**Affiliations:** 1 School of Food Science and Technology, Jiangnan University, Wuxi 214122, China; 2 Key Laboratory of Carbohydrate Chemistry and Biotechnology, Ministry of Education, School of Biotechnology, Jiangnan University, Wuxi 214122, China

**Keywords:** rare sugars, D-allulose, glycerol, aldolase, *ClearColi*

## Abstract

Rare sugars possess potential applications as low-calorie sweeteners, especially for anti-obesity and anti-diabetes. In this study, a fermentation biosystem based on the “DHAP-dependent aldolases strategy” was established for D-allulose and D-sorbose production from glycerol in endotoxin-free *ClearColi* BL21 (DE3). Several engineering strategies were adopted to enhance rare sugar production. Firstly, the combination of different plasmids for *aldO*, *rhaD,* and *yqaB* expression was optimized. Then, the artificially constructed ribosomal binding site (RBS) libraries of *aldO*, *rhaD,* and *yqaB* genes were assembled individually and combinatorially. In addition, a peroxidase was overexpressed to eliminate the damage or toxicity from hydrogen peroxide generated by alditol oxidase (AldO). Finally, stepwise improvements in rare sugar synthesis were elevated to 15.01 g/L with a high yield of 0.75 g/g glycerol in a 3 L fermenter. This research enables the effective production of rare sugars from raw glycerol in high yields.

## 1. Introduction

Rare sugars have become a growing area of interest in the food and pharmaceutical industries because of their special properties and potential applications [1,2,3,4,5]. Among the family of rare sugars, D-allulose is the most well-known sugar and has a variety of physiological functions, such as anti-obesity, anti-hyperglycemia, anti-inflammatory, antioxidant, and neuroprotective effects [6,7,8,9]. D-Allulose was recognized as safe by the United States Food and Drug Administration (FDA) and permitted to be added to a variety of foods and dietary supplements in 2014. Moreover, D-allulose was excluded from the lists of total and added sugars by the US FDA in 2019.

Chemical approaches for D-allulose synthesis suffer from the use of organic reagents, complex byproducts, and tedious purification processes [10,11]. In contrast, D-allulose biosynthesis is becoming more promising. Meanwhile, the Izumoring strategy based on D-allulose 3-epimerase (DAEase) plays a crucial role in D-allulose production from D-fructose [12]. Subsequently, a series of DAEases have been discovered in many microorganisms [13,14,15,16,17,18]. Thus, D-allulose has gradually become one of the most accessible rare sugars. Nevertheless, due to the thermodynamic limitation of the inherent reaction equilibrium, the Izumoring strategy usually provides a low conversion yield and a relatively high production cost. Several efforts have been devoted to improving the conversion rate of D-allulose [19,20,21]. However, these processes incur high expenses and restrict the popularization and application of D-allulose. Recently, a “thermodynamics-driven strategy” for D-allulose bioproduction from low-cost starch was reported [22]. In this biosystem, D-allulose production was performed by multiple enzymes and was thermodynamically favorable, which pushed the overall reactions toward completeness. Nevertheless, expressions and purifications of related enzymes were needed, which was a disadvantage for the industrialization of D-allulose.

Dihydroxyacetone phosphate (DHAP)-dependent aldolases have been used to produce a variety of rare sugars [23,24,25]. Recently, the production of rare sugars from renewable, inexpensive substrates by fermentation has been realized [24,26,27]. In the biosystem, DHAP was generated via the glycolysis pathway or glycerol utilization pathway, followed by the formation of rare sugar-1-phosphates via DHAP-dependent aldolases with the addition D/L-glyceraldehyde as receptors. Finally, rare sugars were produced and released into the fermentation broth by the dephosphorylation of phosphatases. The dephosphorylation step was irreversible, and the theoretical yield of the final product could be up to 100%. Therefore, a fermentation biosystem based on an aldolase strategy provides a promising approach for the biosynthesis of rare sugars.

Recently, we characterized an alditol oxidase from *Streptomyces coelicolor* (*AldO_S. coe_*) and applied it to the generation of the receptor D-glyceraldehyde. Furthermore, whole-cell synthesis of D-allulose and D-sorbose with glycerol as the sole carbon source was achieved [28]. Although the synthesis of D-allulose and D-sorbose was achieved from the sustainable biomass feedstock glycerol, there are three issues that need to be addressed: (1) the host cell *E. coli Rosetta* (DE3) belonging to Gram-negative bacteria contains the molecule lipopolysaccharide (LPS), and LPS (also known as endotoxin) can trigger a pyrogenic response and septic shock may occur; (2) the conversion efficiency for glycerol was relatively low and the total conversion rate of rare sugars from glycerol was only 17.7%; (3) excessive glycerol remaining in the reaction mixture increased the difficulty of further product separation and purification. To address these issues and improve the practicality of rare sugar production, an endotoxin-free *ClearColi* BL21 (DE3) strain was chosen as the host cell in this study, which contains a genetically engineered LPS that does not induce an endotoxic response in human cells [29,30]. Moreover, *ClearColi* BL21 (DE3) has demonstrated its application value for the safe production of recombinant proteins [31,32]. Therefore, an artificial synthetic pathway based on the “DHAP-dependent aldolases strategy” for rare sugar synthesis was redesigned in *ClearColi* BL21 (DE3) to enhance the conversion yield of rare sugars from glycerol. The three core enzymes alditol oxidase (AldO), L-rhamnulose-1-phosphate aldolase (RhaD), fructose-1-phosphatase (YqaB), and another auxiliary peroxidase were used to build a synthetic pathway of rare sugars. After the optimization of the fermentation condition and gene regulation by ribosomal binding site (RBS) libraries, the production yield of rare sugars from 10 g/L glycerol reached 62.9%. The final titer of rare sugars reached 15.01 g/L with a high yield of 0.75 g/g glycerol in a 3 L fermenter. This method enables the effective production of rare sugars from common and cheap starting materials with a high yield.

## 2. Materials and Methods

### 2.1. Chemicals and Reagents

Glycerol was purchased from Greagent (Shanghai, China). Isopropyl-β-D-1-thiogalactopyr-anoside (IPTG) and the antibiotics, including ampicillin, streptomycin, and kanamycin, were all supplied from Sangon Biotech (Shanghai, China). D-Sorbose and D-allulose were purchased from Tokyo Chemical Industry (Tokyo, Japan). All other chemicals were of analytical grade and available from commercial sources.

### 2.2. Plasmids Construction

Bacterial strains, plasmids, and primers involved in this research are listed in Tables S1 and S2, respectively. All DNA manipulations were performed using standard molecular genetic techniques. The plasmids pRSFDuet-*rhaD* and pETDuet-*rhaD* were constructed by amplifying the *rhaD* gene from plasmid pET28a-*rhaD* through *Nco*I and *Xho*I sites. All the recombinant plasmids were verified by sequencing. The plasmid pET28a-*gfp* was constructed by amplifying the *gfp* gene from the plasmid pUC19-*gfp* through *Bam*H I and *Hin*d III sites. The RBS mutant library of *gfp* was constructed by PCR using degenerate primers RBS-*pET28*-F/R (Table S2), followed by phosphorylation and cyclization. The RBS mutant libraries for *rhaD*, *aldO,* and *yqaB* with diverse intensities were constructed according to the above method.

### 2.3. Media and Culture Conditions

*E. coli* DH5α was used for general cloning, and the cells were cultured in LB medium (5 g/L yeast extract, 10 g/L tryptone, and 10 g/L NaCl) at 37 °C, 220 rpm. The antibiotics ampicillin (100 μg/mL), streptomycin (50 μg/mL), and kanamycin (50 μg/mL) were added where appropriate. The endotoxin-free strain *ClearColi* BL21 (DE3) (Lucigen, Madison, WI, USA) was used as the chassis strain for rare sugar production. The engineered strains were grown in LB medium overnight as the seed culture. Then 1% inoculum of the seed was transferred to a 250 mL flask containing 50 mL of TB medium (24 g/L yeast extract, 12 g/L tryptone, 16.4 g/L K_2_HPO_4_·3H_2_O, and 2.31 g/L KH_2_PO_4_). All recombinant strains were cultured at 37 °C and 220 rpm. When the optical density (OD) at 600 nm was 0.6–0.8, 0.1 mM IPTG and 10 g/L glycerol with final concentrations were added and cultured for another 36 h at 30 °C. For high-throughput screening, a single colony was randomly selected and cultured in 96-well deep plates containing 1 mL of LB medium with the corresponding antibiotic at 37 °C and 180 rpm for 12 h. Then 20 μL of the cell culture was inoculated with 2 mL of fresh TB medium in 24-well plates. After cultivation at 37 °C for 5 h, 0.1 mM IPTG and 10 g/L glycerol with final concentrations were added and cultured for another 48 h at 30 °C for rare sugar production. For fermentation, 20 mL of seed liquid from the engineered strain grown in LB medium at 37 °C and 220 rpm for 12 h was transferred to a 3 L fermenter (T&J-Atype, Shanghai, China) containing 1.0 L of TB medium with the corresponding antibiotic. When OD_600_ reached around 1.0, 0.1 mM IPTG and 10 g/L glycerol with final concentrations were added to induce the gene expression. After induction for 24 h, 10 g/L of glycerol was supplemented. The culture conditions were maintained as follows: 30 °C, airflow rate of 2.0 vvm, and a stirring speed of 400 rpm. The pH value was automatically controlled at 7.5 with 4.0 mol/L NH_3_·H_2_O.

### 2.4. Construction of RBS Library of gfp Gene

The sequence (ATATACC) of RBS from pET28a was mutated with saturation primers (RNNNNNN, where R means A/G and N means A/T/C/G). The plasmids with the RBS mutation of *gfp* were digested by *Dpn* I at 37 °C for 1 h. After purification with a PCR product kit, the RBS mutant plasmids were transformed into *ClearColi* BL21 (DE3) to obtain the RBS mutant library of *gfp*. For the high-throughput screening, the single colonies were cultured as described above. Cells with GFP expression were harvested and washed three times with PBS. The RBS intensity was evaluated by measuring the fluorescence of reporter GFP. The fluorescent intensity (FI) of the diluted solution was detected by a multi-mode reader (Biotek/H1, Winooski, VT, USA) in a 96-well microtiter plate (black with a transparent bottom, ThermoFisher, Waltham, MA, USA) with an excitation at 395 nm and emission at 509 nm. The cell growth was assayed by detecting the value of OD_600_ with a UV-1700 spectrophotometer (Shimadzu, Kyoto, Japan). The *FI* was calculated using the following formula:

$$FI=\frac{Fluorescence}{OD}$$

### 2.5. Construction of RBS Libraries of rhaD, aldO, and yqaB Genes

The RBS sequence upstream of *rhaD* was mutated by PCR with saturation primers (*RBS-pET28*-F/R) using plasmid pET28a-*rhaD* as the template. Then, the plasmids with the RBS mutation upstream of *rhaD* were digested by *Dpn* I at 37 °C for 1 h. After purification, the plasmids harboring mutated RBS sequences of *rhaD* were co-transformed with the plasmid pCDFDuet-*yqaB*-*aldO* into *ClearColi* to obtain the RBS mutation library of the *rhaD* gene. The RBS mutation libraries of *aldO* and *yqaB* genes were constructed with similar operations. The mutant colonies were randomly picked and grown in LB medium. According to the above high-throughput screening process, the culture supernatant containing rare sugars was spotted on TLC silica gel (20 × 20, Merck, Darmstadt, Germany) and stained with anisaldehyde staining. Soon afterwards, the TLC plates were heated until green spots appeared. The strains with a darker green color compared with the wild-type strain were selected during the primary screening and then further verified by HPLC in the second screening. The RBS mutant plasmids of *rhaD* were co-transformed with the RBS mutant plasmids of *aldO* into *ClearColi* to obtain the RBS mutation libraries of *rhaD* and *aldO* genes. Finally, the RBS mutant sequence of the highest strain was confirmed by Tianlin Biotechnology (Wuxi, China).

### 2.6. Analytical Method

The protein expression was detected by 12% (*v*/*v*) sodium dodecyl sulfate polyacrylamide gel electrophoresis (SDS-PAGE), and the gel was stained with Coomassie Brilliant Blue. The amounts of glycerol and rare sugars in the culture supernatant were determined by high-performance liquid chromatography (HPLC) [28].

## 3. Results and Discussion

### 3.1. Construction of a Biosynthetic Pathway for Rare Sugar Production from Glycerol

**Figure 1:**
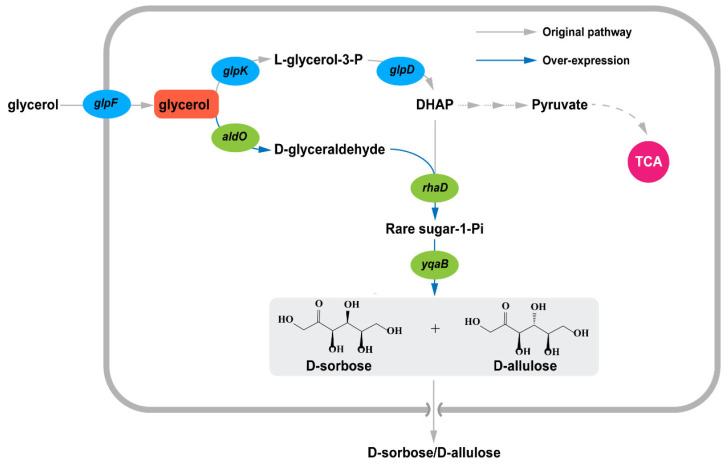
Construction of synthetic pathway for rare sugar production from glycerol in *ClearColi*.

**Figure 2:**
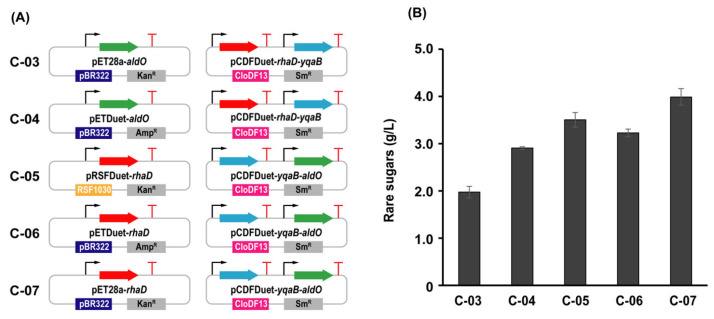
Effects of different overexpression manners for related enzymes on the production of rare sugars. (**A**) Schematic diagram of different plasmid combinations. (**B**) Production of rare sugars using different plasmid combinations.

The Izumoring strategy is widely adopted for rare sugar production. However, due to the thermodynamic limitation of the inherent reaction equilibrium, the maximum conversion rate for D-allulose from D-fructose is approximately 30%. As a complementary alternative, we previously constructed an *E. coli* whole-cell platform for cascade synthesis of rare sugars based on L-rhamnulose-1-phosphate aldolase [28]. Although the synthesis of D-allulose and D-sorbose was achieved from the sustainable biomass feedstock glycerol, the total conversion rate was also low. To improve practicality, an artificial synthetic pathway for rare sugar synthesis was redesigned in a *ClearColi* strain without endotoxin. Naturally, glycerol can be utilized by *ClearColi* as the sole carbon source, which is then converted into intracellular DHAP through the original metabolic pathway. Meanwhile, D-glyceraldehyde can be generated from glycerol by overexpressing AldO [33,34,35]. The condensation reaction of DHAP and D-glyceraldehyde is catalyzed by RhaD to obtain the corresponding rare sugar-1-phosphate, which is further dephosphorylated to afford D-sorbose and D-allulose by YqaB phosphatase (Figure 1). In order to verify the feasibility of rare sugar production, the preliminary fermentation experiments using the recombinant strains C-01 overexpressing AldO, C-02 overexpressing RhaD and YqaB, and C-03 overexpressing AldO, RhaD, and YqaB were tested. The results showed only the C-03 strain could produce 1.97 g/L of rare sugars (D-sorbose and D-allulose) with a yield of 0.20 g/g glycerol (Figure S1). To study the effects of different overexpression methods on the efflux of rare sugars during glycerol fermentation, five recombinant strains containing different combinations of plasmids were examined individually. As a result, the strain C-07 exhibited the highest yield and could produce 3.98 g/L of rare sugars (D-sorbose and D-allulose) after fermentation, which was approximately 2.0-fold than that of C-03 (Figure 2). Meanwhile, the protein expression levels of strain C-07 were verified by SDS-PAGE (Figure S2). To further improve the yield, several fermentation conditions, including medium, induction OD_600_, pH value, and culture temperature were investigated. The results showed that the optimal fermentation conditions were TB medium, an induction OD_600_ of 1.2, a pH value of 7.5, and a culture temperature of 30 °C (Figure S3). The rationale for using NH_3_·H_2_O to control the pH value is that ammonia water can provide a nitrogen source that can be rapidly utilized and a stable pH buffer for the strain’s growth. After the optimization, the total concentration of rare sugars reached 4.45 g/L with a yield of 0.45 g/g glycerol.

### 3.2. Characterization of Strength Variations of GFP Based on the RBS Library

**Figure 3:**
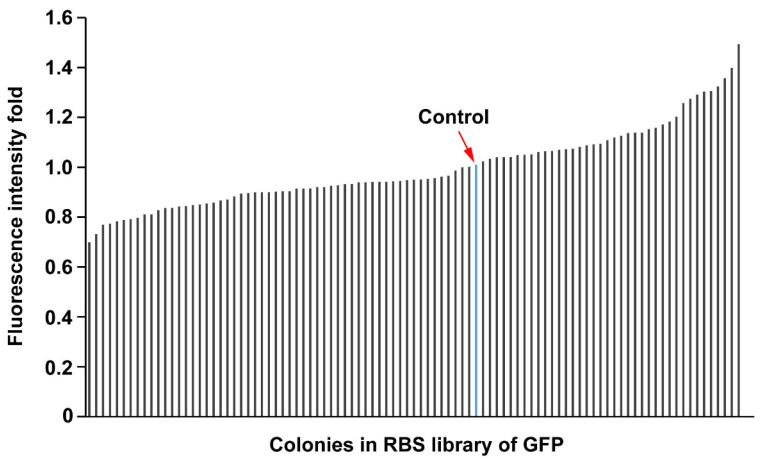
Evaluation of the strength variations of GFP after modulating the *gfp* gene with the RBS library.

In general, regulating the expression and activities of the enzymes in the pathway contributes to the accumulation of the target product [36]. RBS regulation has become a useful tool for fine-tuning the expression of the enzyme in synthetic biology [37,38]. To test the strength variations of GPF, the RBS sequences in front of the *gfp* gene were changed based on the primer pair of RBS-*pET28*-F/R (Figure S4). After induction, the fluorescence level of GPF was determined by a multi-mode reader (Figure 3). Among the 97 colonies selected randomly, the strengths of the RBS varied from 0.7 to 1.6 times compared with that of the wild-type strain. These results indicated that the RBS library had a relatively broad range of strength variations.

### 3.3. Modulating aldO, rhaD, and yqaB Genes with the RBS Library

**Figure 4:**
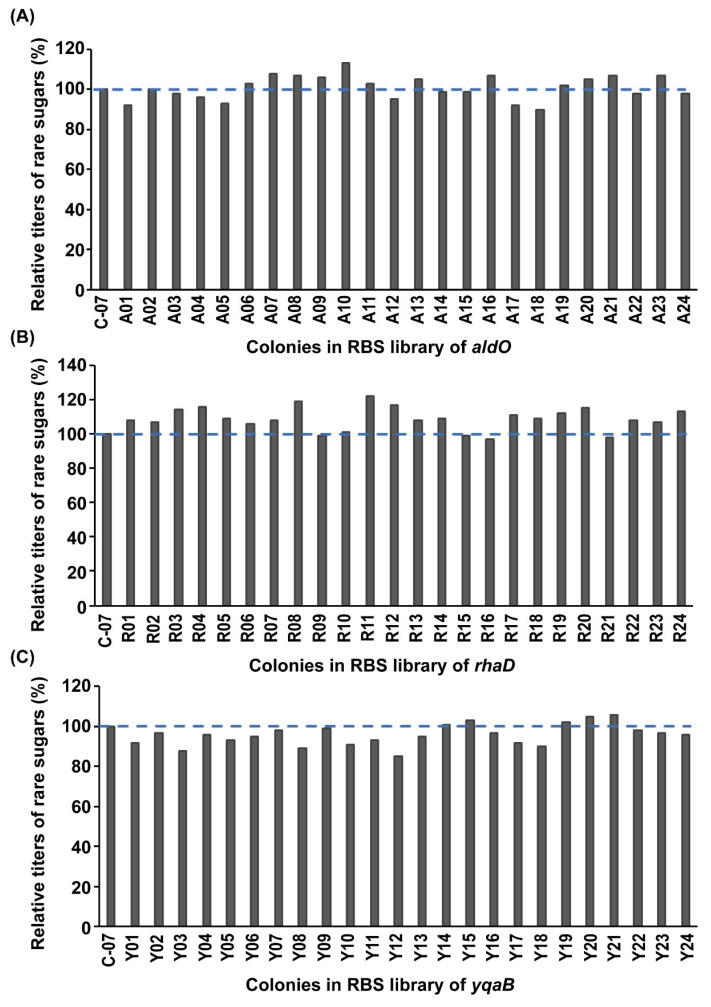
Rare sugar titer detection after modulating *aldO, rhaD,* and *yqaB* genes individually with the RBS library. (**A**) Modulating *aldO* with the RBS library. (**B**) Modulating *rhaD* with the RBS library. (**C**) Modulating *yqaB* with the RBS library. The blue dashed line represents the relative titer of rare sugars for the control without RBS modulation.

In order to improve the yield of rare sugars, the expressions of *aldO, rhaD,* and *yqaB* were further optimized individually by RBS engineering, and 24 colonies out of a total of 382 colonies in each group were selected in primary screening (Figure 4). The relative titers of rare sugars analyzed by HPLC are shown in Figure 4. After *aldO* regulation with the RBS mutation, strain A10 had the highest titer of rare sugars, which increased by 13% compared with that of strain C-07 (Figure 4A). After *rhaD* regulation with the RBS mutation, strain R11 exhibited the highest titer of rare sugars, which increased by 22% compared with that of strain C-07 (Figure 4B). After *yqaB* regulation with RBS mutation, strain Y21 demonstrated the highest titer of rare sugars, which increased by 6% compared with that of strain C-07 (Figure 4C). These results indicated that the RBS library regulating the above three key genes was more conducive to the synthesis of rare sugars. The RBS sequences of the strains with the highest titers are shown in Table 1.

### 3.4. Dual Modulating aldO and rhaD Genes with the RBS Library

After modulating *aldO, rhaD,* and *yqaB* genes with the RBS library individually, the above genes displayed different effects on the production of rare sugars. Their influence orders were as follows: *rhaD* > *aldO* > *yqaB*, which indicated that RhaD was the main rate-limiting enzyme, which was consistent with the previous results [35].

**Figure 5:**
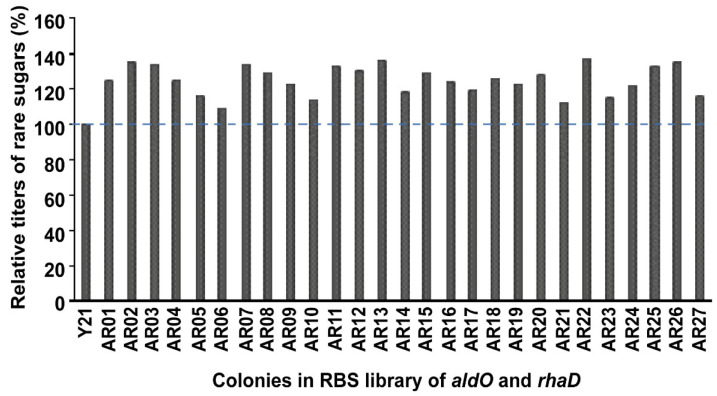
Rare sugar titer detection after dual modulating *aldO* and *rhaD* with RBS libraries. The blue dashed line represents the relative titer of rare sugars for the control (strain Y21).

**Figure 6:**
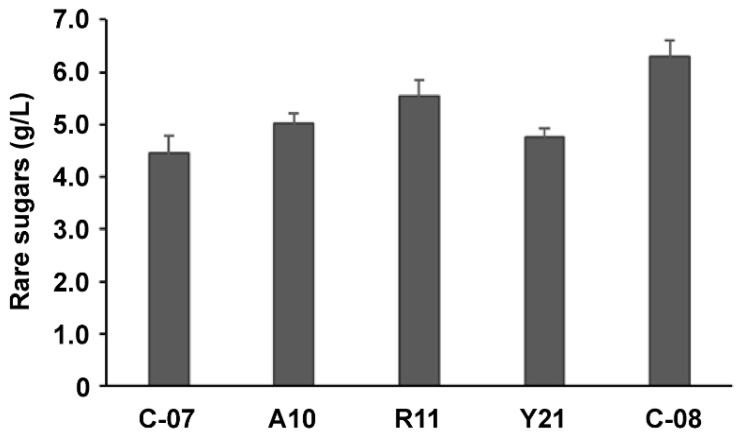
Quantification of rare sugar production for the representative strains after modulating with RBS libraries.

To enhance the titer of rare sugars, the expression levels of these three key genes in the pathway should be well balanced. Therefore, the dual modulation of *aldO* and *rhaD* genes with the RBS library was further carried out to improve rare sugar production based on strain Y21, and 27 colonies out of a total of 960 colonies were selected and confirmed by HPLC. The strain AR22 (renamed C-08) exhibited the highest titer of rare sugars, and the titer increased by 33% compared with that of strain Y21 (Figure 5). It suggested that combinatorial regulation with the RBS library was more effective than individual regulation. The RBS sequences of the strain C-08 are also shown in Table 1. Soon afterwards, the above strains with the highest titers were selected to be tested three times. These repetitive results were consistent with the primary screening results (Figure 6). As a result, the titer of rare sugars reached 6.29 g/L with a yield of 0.63 g/g glycerol.

### 3.5. Effects of Peroxidases Overexpression on the Production of Rare Sugars

**Figure 7:**
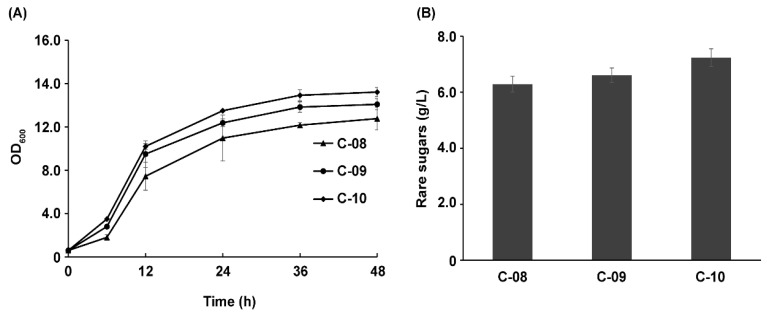
Effect of peroxidase overexpression on the production of rare sugars. (**A**) Effects of peroxidase overexpression on the growth of recombinant strains. (**B**) Production of rare sugars using the recombinant strains overexpressing peroxidases.

Peroxidase overexpression would eliminate the damage or toxicity from hydrogen peroxide generated by AldO, which is beneficial for cell growth and the accumulation of rare sugars [28,39,40]. Therefore, two different peroxidases encoded by *KatE* and *prx02* from *E. coli* MG1655 were also overexpressed. The growth curves displayed in Figure 7A showed that the strain overexpressing KatE (strain C-09) or Prx02 (strain C-10) increased cell growth. However, overexpression of KatE or Prx02 only slightly improved the total production titer of rare sugars, as shown in Figure 7B. The strain C-10 showed the highest titer of rare sugars, and the production of rare sugars reached 7.23 g/L with a high yield of 0.72 g/g glycerol.

### 3.6. Evaluation of Rare Sugar Production by the Engineered Strain in a 3-L Fermenter

**Figure 8:**
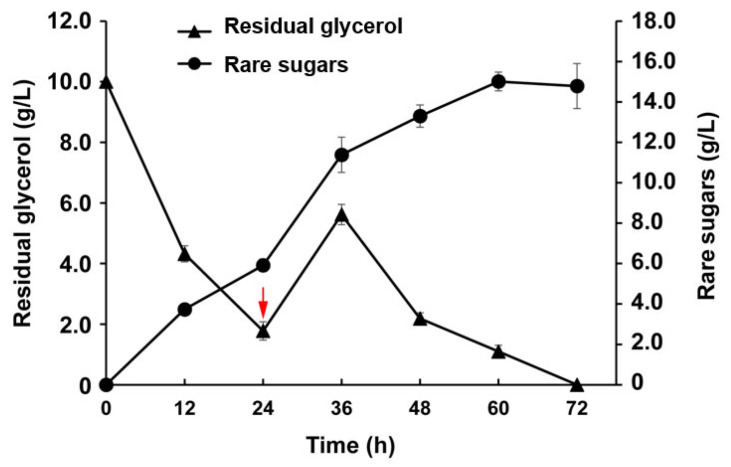
Time profiles of rare sugars and residual glycerol in the engineered strain. The red arrow was the point of supplementation with 10 g/L of glycerol.

To further characterize *ClearColi* strain C-10 and investigate its potential application for rare sugar production on a large scale, the fermentation was performed in a 3-L fermenter. As shown in Figure 8, rare sugars were steadily produced, and glycerol was consumed continually. After induction for 24 h, the concentration of residual glycerol in the medium decreased below 2 g/L. To improve the production yield of rare sugars, a strategy with a glycerol supplement was employed. To our delight, the amount of rare sugars with a rapid increase was obviously observed after the glycerol supplement (Figure 8). As a result, 15.01 g/L of rare sugars (D-sorbose/D-psicose = 1.2/1) were produced with a productivity of 0.25 g/L/h at 60 h during the entire fermentation. The highest yield of rare sugars reached 0.75 g/g glycerol, and the glycerol in the medium was completely consumed when the fermentation was finished. To further achieve the efficient biosynthesis of rare sugars, a variety of metabolic engineering strategies, such as dynamic regulation to balance the flux between growth and products in the metabolic network, will be adopted in the future.

## 4. Conclusions

It is feasible to achieve the goal of the conversion of glycerol to rare sugars with a high yield by fermentation. In this study, the “DHAP-dependent aldolases strategy” was adopted for D-allulose and D-sorbose biosynthesis from glycerol. After regulating the three key genes in the pathway individually or combinatorically with the RBS library, the titers of rare sugars were both improved compared to the original strain. Meanwhile, the combinatorial regulation of two genes with the RBS library was more effective than the individual regulation. After RBS regulation strategies were implemented, the final titers of rare sugars reached 15.01 g/L with a high yield of 0.75 g/g glycerol in a 3-L fermenter with a glycerol supplement. These results exceed the best data previously reported for D-allulose and D-sorbose from glycerol. Taken together, this fermentation biosystem will remarkably simplify the production procedure of rare sugars and contribute to their cost-effective manufacture.

## Figures and Tables

**Table 1:** RBS sequences of regulation genes in the representative strains.

Strain	Gene	RBS Sequence
A10	*aldO*	CAGGAGGTAGCCG
R11	*rhaD*	CAGGAGGCATTTC
Y21	*yqaB*	CAGGAGGCATTTC
C-08	*aldO*	CAGGAGAAACAAC
	*rhaD*	CAGGAGGTTGTTA

## Data Availability

The date are available from the corresponding authors.

## Supplementary Materials

The following supporting information can be downloaded at: https://www.mdpi.com/article/10.3390/foods12163078/s1. Table S1: Strains and plasmids (Refs [28,41]); Table S2: Primers; Figure S1–S3: experimental results; Figure S4: RBS library construction.
