# Supplementary material for: Highly Efficient Synthesis of Rare Sugars from Glycerol in Endotoxin-Free ClearColi by Fermentation
Source: Foods. 2023 Aug 16;12(16):3078. doi: 10.3390/foods12163078 (PMC10453619; doi:10.3390/foods12163078)
Supplement: Supplementary file 1 [file foods-12-03078-s001.zip › foods-2524364-supplementary.pdf]

Supporting Information

Highly efficient synthesis of rare sugars from glycerol in endotoxin-free *ClearColi* by fermentation

Yahui Gao [1], Zhou Chen [2], Hideki Nakanishi [2], Zijie Li [2 *]

[1] School of Food Science and Technology, Jiangnan University, Wuxi, Jiangsu 214122, China

[2] Key Laboratory of Carbohydrate Chemistry and Biotechnology, Ministry of Education, School of Biotechnology, Jiangnan University, Wuxi, Jiangsu 214122, China

*Corresponding author at Key Laboratory of Carbohydrate Chemistry and Biotechnology, Ministry of Education, School of Biotechnology, Jiangnan University, Wuxi, 214122, China

E-mail address: lizijie@jiangnan.edu.cn (Z. Li).

Table S1 Strains and plasmids used in this study

**Strains or Plasmids**	**Characteristics**	**Source or Reference**
E. coli DH5α	*endA1 supE44 recA1 gyrA96 relA1 deoRU169 Φ 80dlacZ ΔM15 mcrAΔ(mrr-hsdRMS-mcrBC)*	Invitrogen
ClearColi BL21 (DE3)	*Omp Thsd ThsdS (rB-mB-) gal dcm lon λ(DE3) msb148ΔgutQΔkdsDΔlpxLΔlpxMΔpagPΔlpxPΔeptA*	Lucigen
C-01	*ClearColi* containing plasmid pET28a-*aldO*	This study
C-02	*ClearColi* containing plasmid pCDFDuet-*rhaD-yqaB*	This study
C-03	*ClearColi* containing plasmid pCDFDuet-*rhaD-yqaB* and pET28a-*aldO*	This study
C-04	*ClearColi* containing plasmid pCDFDuet-*rhaD-yqaB* and pETDuet-*aldO*	This study
C-05	*ClearColi* containing plasmid pCDFDuet-*yqaB-aldO* and pRSFDuet-*rhaD*	This study
C-06	*ClearColi* containing plasmid pCDFDuet-*yqaB-aldO* and pETDuet-*rhaD*	This study
C-07	*ClearColi* containing plasmid pCDFDuet-*yqaB-aldO* and pET28a-*rhaD*	This study
C-08	*ClearColi* containing plasmid pCDFDuet-RBS_{opt}-*yqaB*-RBS_{opt}-*aldO* and pET28a-RBS_{opt}-*rhaD*	This study
C-09	*ClearColi* containing plasmid pCDFDuet-RBS_{opt}-*yqaB*-RBS_{opt}-*aldO-katE* and pET28a-RBS_{opt}-*rhaD*	This study
C-10	*ClearColi* containing plasmid pCDFDuet-RBS_{opt}-*yqaB*-RBS_{opt}-*aldO-prx02* and pET28a-RBS_{opt}-*rhaD*	This study
Plasmids		
pET28a-*gfp*	pET28a carrying the gene *gfp*	In our lab
pET28a-*rhaD*	pET28a carrying the gene *rhaD*	In our lab
pRSFDuet-*rhaD*	pRSFDuet-1 carrying the gene *rhaD*	This study

pETDuet-*rhaD*	pETDuet-1 carrying the gene *rhaD*	This study
pCDFDuet-*rhaD*-*yqaB*	pCDFDuet-1 containing the genes *rhaD* and *yqaB*	[41]
pET28a-*aldO*	pET28a carrying the gene *aldO*	[28]
pETDuet-*aldO*	pETDuet-1 carrying the gene *aldO*	This study
pCDFDuet-*yqaB-aldO*	pCDFDuet-1 carrying the genes *yqaB* and *aldO*	[28]
pETDuet-*yqaB-aldO*	pETDuet-1 carrying the genes *yqaB* and *aldO*	This study
pCDFDuet-RBS_{opt}-*yqaB*-RBS_{opt}-*aldO-katE*	pCDFDuet-1 carrying the genes *yqaB*, aldO and *prx02* with corresponding optimal RBS	This study
pCDFDuet-RBS_{opt}-*yqaB*-RBS_{opt}-*aldO-prx02*	pCDFDuet-1 carrying the genes *yqaB*, aldO and *katE* with corresponding optimal RBS	This study

Table S2 Primers used in this study

Primer name	Nucleotide sequence (5'-3')
RBS-pET28-F	TAATTTTGTTTAACTTTAAGCAGGAGRNNNNNNATGGGCAGCAGCCATCATCA
RBS-pET28-R	TGATGATGGCTGCTGCCCATNNNNNNYCTCCTGCTTAAAGTTAAACAAAATTA
RBS-aldO-F	GTATATTAGTTAAGTATAAGCAGGAGRNNNNNNATGGCTAGCATGACTGGTGG
RBS-aldO-R	CCACCAGTCATGCTAGCCATNNNNNNYCTCCTGCTTATACTTAACTAATATAC
RBS-yqaB-F	TAATTTTGTTTAACTTTAAGCAGGAGRNNNNNNATGGTGTACGAGCGTTATGC
RBS-yqaB-R	GCATAACGCTCGTACACCATNNNNNNYCTCCTGCTTAAAGTTAAACAAAATTA
katE-F	AAGCTTGCGGCCGCAAAGGAGATATAATGTCGCAACATAACGAAAA
katE-R	GGTTTCTTTACCAGACTCGAGTCAGGCAGGAATTTTGTCAAT (*Xho*I)
prx02-F	AAGCTTGCGGCCGCAAAGGAGATATAATGTCTCAGGTTCAGAGTGGCA
prx02-R	GGTTTCTTTACCAGACTCGAGTTACAGCGCCATCAACTTGTCC (*Xho*I)

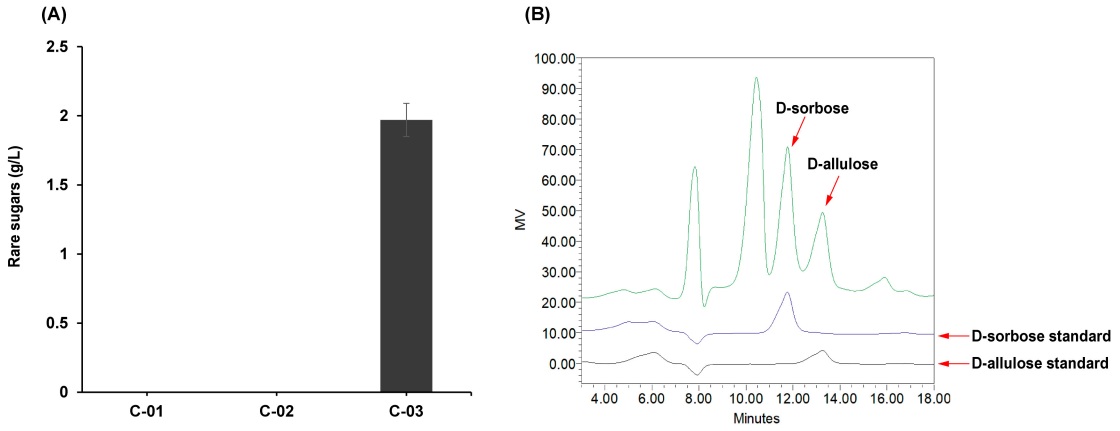

Figure S1. (A,B) Production of D-allulose and D-sorbose was quantified by HPLC

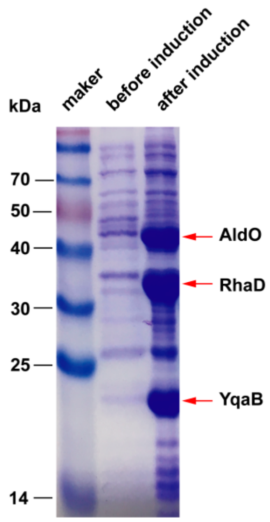

Figure S2. The protein expression level of the strain C-07 was detected by SDS-PAGE.

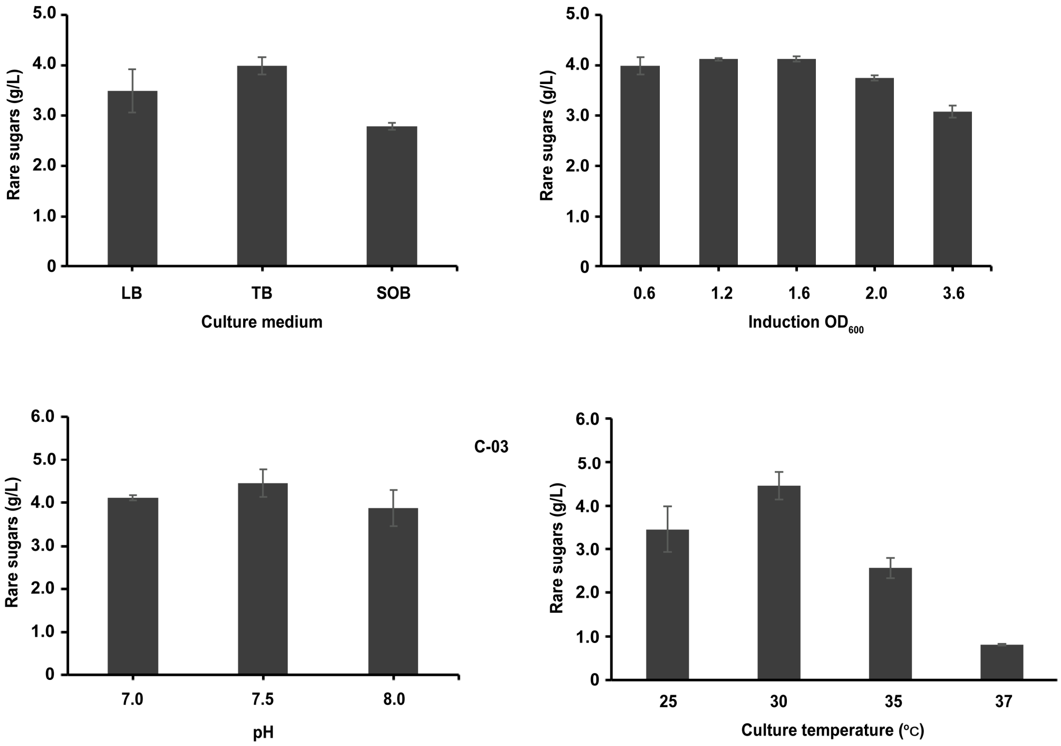

Figure S3. Optimization of fermentation conditions

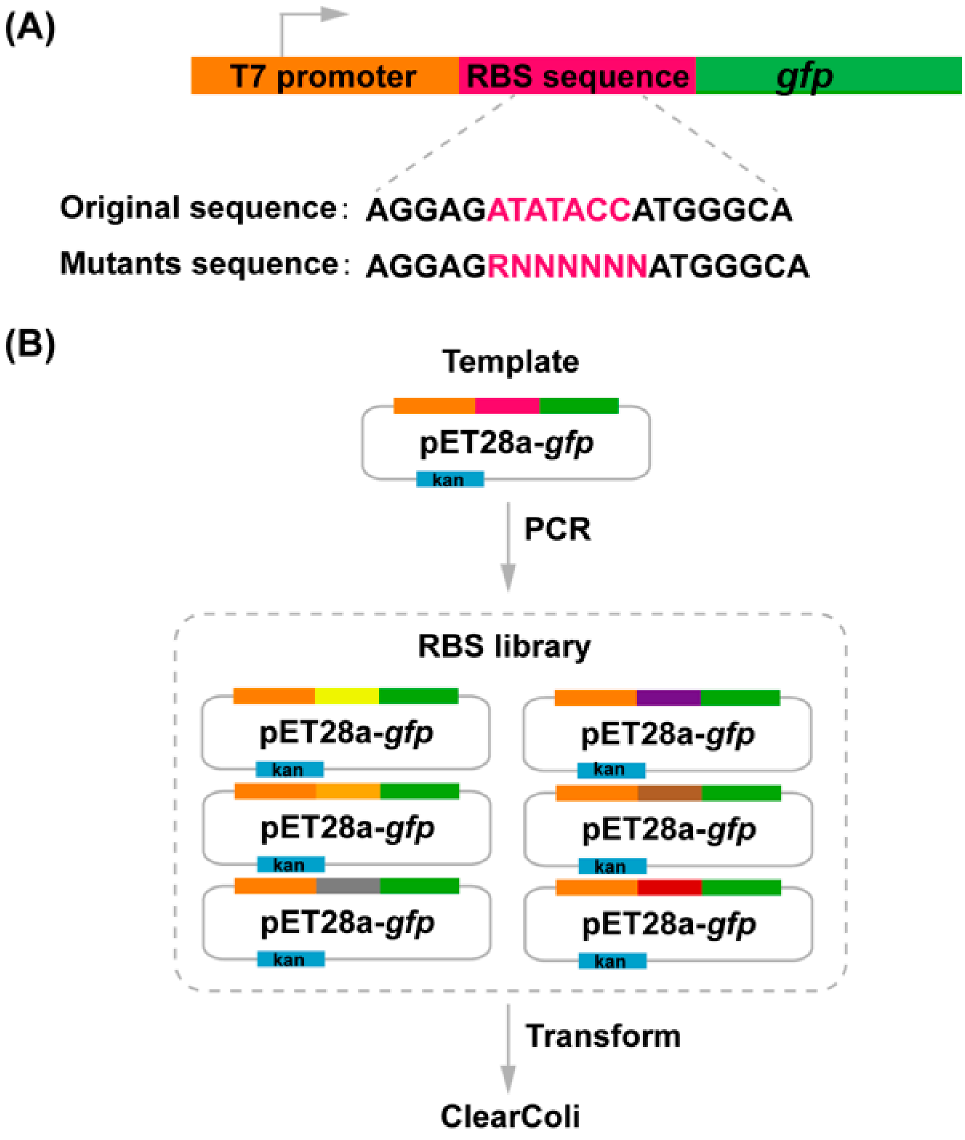

Figure S4. (A,B) Construction of the RBS library for GFP

References

41. Li, Z., B. He, Y. Gao and L. Cai. "Synthesis of D-sorbose and D-psicose by recombinant *Escherichia coli.*" *Journal of Carbohydrate Chemistry* 34 (2015): 349-357.

28. Chen, Z., Z. Li, F. Li, N. Wang and X.-D. Gao. "Characterization of alditol oxidase from streptomyces coelicolor and its application in the production of rare sugars." *Bioorganic & Medicinal Chemistry* 28 (2020): 115464.
